# Molecular characterization and targeted therapeutic approaches in breast cancer

**DOI:** 10.1186/s13058-015-0560-9

**Published:** 2015-04-23

**Authors:** Angela Toss, Massimo Cristofanilli

**Affiliations:** Department of Oncology, Haematology and Respiratory Diseases, University of Modena and Reggio Emilia, Via del Pozzo 71, Modena, 41125 Italy; Department of Medical Oncology, Jefferson University Hospital, 1100 Walnut Street, Philadelphia, PA 19107 USA

## Abstract

Despite the wide improvements in breast cancer (BC) detection and adjuvant treatment, BC is still responsible for approximately 40,000 deaths annually in the United States. Novel biomarkers are fundamental to assist clinicians in BC detection, risk stratification, disease subtyping, prediction of treatment response, and surveillance, allowing a more tailored approach to therapy in both primary and metastatic settings. In primary BC, the development of molecular profiling techniques has added prognostic and predictive information to conventional biomarkers - estrogen receptor, progesterone receptor, and human epidermal growth factor receptor 2. Moreover, the application of next-generation sequencing and reverse-phase protein microarray methods in the metastatic setting holds the promise to further advance toward a personalized management of cancer. The improvement in our understanding on BC biology associated with the study of the genomic aberrations characterizing the most common molecular subtypes allows us to explore new targets for drug development. Finally, the integration of cancer stem cell-targeted therapies and immune therapies in future combination regimens increases our chances to successfully treat a larger proportion of women with more aggressive and resistant metastatic disease. This article reviews the current state of novel biological markers for BC, the evidence to demonstrate their clinical validity and utility, and the implication for therapeutic targeting.

## Introduction

Breast cancer (BC) represents the most common cancer among women; there were 232,670 estimated new cases and 40,000 estimated deaths in the United States in 2014 [1]. In recent decades, the widespread use of mammographic screening has increased the rate of regional disease detection, and the development of more effective adjuvant chemotherapeutic regimens, extended use of endocrine therapies, and standard application of targeted agents have all contributed to improve outcomes of women with primary BC. However, the widespread application of these diagnostic and therapeutic interventions requires significant resources and is associated with treatment-related morbidity; therefore, determining the subgroup of patients who can truly benefit from the implementation of such advanced measures is still a challenge.

For years, researchers have investigated clinical tools and molecular approaches with the aim of discovering a combination of clinical and biological features that could predict cancer features and behavior, allowing a more tailored approach to therapy. New biomarker development is fundamental to assist clinicians in BC detection and diagnosis, risk stratification, disease subtyping, prediction of treatment response, and surveillance, allowing a personalized cancer management. The integration between novel biomarkers and routinely tested clinic-pathological features, such as hormone receptor (HR) and human epidermal growth factor receptor 2 (HER2) status, may guide clinicians in systemic therapy decisions in both primary and metastatic settings. This article reviews the current state of novel biological markers for BC, the evidence to demonstrate their clinical validity and utility, and the implication for therapeutic targeting.

## Breast cancer subtypes and gene expression profile tests

From the clinical point of view, BC can be classified according to the immunohistochemistry/fluorescence *in situ* hybridization (IHC/FISH) profile and divided on the basis of the presence of estrogen receptor (ER), progesterone receptor (PR), and HER2.

At the molecular level, Perou and colleagues [2] analyzed BC gene expression patterns derived from cDNA microarrays, initially identifying four major intrinsic gene signatures: luminal, HER2-enriched, basal-like, and normal breast-like subtype. Subsequent studies led to the division of luminal tumors in two subgroups (luminal A and luminal B) and demonstrated a correlation between these gene expression patterns and survival, disease relapse, site of metastasis, and chemotherapy response [3-5]. Over the years, other molecular subtypes have been described, such as *claudin low* and molecular apocrine tumors. In 2009, Parker and colleagues [6] developed an efficient 50-gene classifier, called Prediction Analysis of Microarray (PAM50), that reanalyzed the previous five subgroups defining the four major intrinsic subtypes currently known: luminal A, luminal B, HER2-enriched, and basal-like (Table 1).

**Table 1:** **Breast cancer intrinsic subtypes with prevalent immuno-histochemical profiles and options of treatment** [3]

**Intrinsic subtype**	**cDNA microarrays**	**IHC**	**Treatment**
Luminal A	The highest expression of the ER α gene, GATA-binding protein 3, X-box-binding protein 1, trefoil factor 3, hepatocyte nuclear factor 3 α, and estrogen-regulated LIV-1	ER- and/or PR-positive HER2-negative Ki-67 < 14%	Endocrine therapy (chemotherapy for selected patients)
Luminal B	Low to moderate expression of the luminal-specific genes, including the ER cluster	ER- and/or PR-positive HER2-negative with Ki-67 ≥ 14%	Endocrine therapy ± chemotherapy
		ER- and/or PR-positive HER2-positive with any Ki-67	Chemotherapy + anti-HER2 therapy + endocrine therapy
HER2-enriched	High expression of several genes in the ERBB2 amplicon at 17q22.24, including ERBB2 and GRB7	ER- and PR-negative HER2-positive	Chemotherapy + anti-HER2 therapy
Basal-like	High expression of keratins 5 and 17, laminin, and fatty acid-binding protein 7	ER- and PR-negative HER2-negative	Chemotherapy

ER, estrogen receptor; HER2, human epidermal growth factor receptor 2; IHC, immunohistochemistry; PR, progesterone receptor.

In recent years, five novel gene expression prognostic tests for BC have been developed: MammaPrint, MapQuant Dx, Oncotype DX, PAM50, and Theros Breast Cancer Index. The rationale for developing multi-gene-based prognostic tests is not only to add prognostic and predictive information to conventional biomarkers but to provide more reliable and reproducible techniques than the IHC-based assays, reducing technical errors and subjective interpretation [7]. One of the first commercially available and US Food and Drug Administration (FDA)-approved signatures was the 70-gene MammaPrint assay, which stratifies patients into low- or high-risk for distant metastases at 5 years. More recently, the 21-gene Oncotype DX assay was developed to estimate the risk of relapse in ER^+^, node-negative BC and their chemo-sensitivity. Oncotype DX divides patients into three groups on the basis of their recurrence score (RS): low-risk (RS of less than 18), intermediate-risk (RS of 18 to 30), or high-risk (RS of more than 31) [8]. As previously described, the PAM50 test defined the four major intrinsic subtypes of BC through the analysis of 50 classifier genes and five control genes. Along with the identification of subtypes, PAM50 has been shown to be an independent predictor of survival in BC [9]. PAM50 generates a numerical score (risk of recurrence, or ROR) that along with clinical features estimates the risk of relapse at 10 years in postmenopausal women with stage I/II node-negative or stage II node-positive (one to three positive lymph nodes) and HR-positive BC [10].

In patients with ER-positive and node-negative early BC, the PAM50 platform has been demonstrated to provide more prognostic information than the Oncotype DX test, since PAM50 was better able to distinguish between intermediate- and high-risk patients [11]. On these bases, the platform was recently cleared by the FDA. Notably, in the same study, PAM50 and Oncotype DX assays were compared with the IHC4 score. IHC4 is a prognostic model that combines quantitative IHC measures of ER, PR, HER2, and Ki-67 performed in high-quality laboratories. In this study, relatively similar information was provided by ROR and IHC4 in all patients, but in the HER2-negative/node-negative subgroup, ROR was more informative than IHC4 [11].

The other multi-gene-based assays showed similar prognostic performances, and their prognostic value is due mainly to the ability of providing a robust measurement of proliferation activity, particularly in ER^+^/HER2^−^ patients [12]. However, only the Oncotype Dx assay achieved level IB evidence and has been incorporated into current National Comprehensive Cancer Network and American Society of Clinical Oncology guidelines, since it demonstrated a role as a predictive test in two prospectively designed retrospective studies with tumor specimens obtained from randomized clinical trials comparing tamoxifen with or without chemotherapy [13]. The evaluation and the comparison between the predictive value of PAM50 and Oncotype DX are ongoing in the prospective RxPONDER trial (NCT01272037), and results from the prospective MINDACT (NCT00433589) and TAILORx (NCT00310180) trials are awaited to have direct evidence of the predictive value in the adjuvant setting of, respectively, MammaPrint and Oncotype DX assays.

Through multi-gene profiling tools, more information can be obtained from tumor tissues. For instance, the PAM50 platform provides quantitative values for proliferation, luminal gene expression, estrogen receptor 1 (*ESR1*), progesterone receptor gene (*PGR*), and *HER2*. Moreover, on the same tissue used for MammaPrint, the BluePrint test can be performed. BluePrint is a molecular subtyping assay that analyzes the mRNA levels of 80 additional genes to better discriminate among the molecular subtypes. Combining MammaPrint and BluePrint allows patients to be stratified into luminal-type/MammaPrint low-risk (similar to luminal A), luminal-type/MammaPrint high-risk (similar to luminal B), HER2-type, and basal-type [14]. This stratification has demonstrated several implications in neoadjuvant trials. The recent CTNeoBC pooled analysis of 12 neoadjuvant randomized trials highlighted that patients who achieve a pathologic complete response (pCR) show more favorable outcomes. Notably, the achievement of pCR varied according to BC subtype, and the prognostic value was greatest in more aggressive subtypes. In particular, the association between pCR and long-term outcomes was strongest in patients with HR^+^/HER2^−^/grade 3 tumors, triple negative breast cancer (TNBC), and HR^−^/HER2^+^ who received trastuzumab [15]. Interestingly, Glück and colleagues [16] investigated the correlation between pCR rate after neoadjuvant chemotherapy (NACT) and long-term outcome comparing BluePrint and MammaPrint combined model versus clinical subtyping using IHC/FISH. This study confirmed the notable benefit in response to neoadjuvant treatment, and thus in the long-term outcome, of patients with HER2-type and basal-type. Overall, BluePrint with MammaPrint molecular subtyping was shown to improve the stratification of patients in the neoadjuvant setting, improving prognostic estimation versus IHC/FISH [16]. Thus, molecular signatures provide a more accurate representation of BC biological features, allowing prognostication at the time of initial diagnosis, prediction of benefit from adjuvant therapy, and response to NACT (Table 2). Nevertheless, despite their demonstrated efficacy, there are still large geographic differences in the adoption of these tests, probably reflecting variations in economies, health systems, and physician training. Therefore, in many institutions, clinical and immunohistochemical evaluation remains the reference method for the classification of BC.

**Table 2:** **Main features of the principal available multi-gene assays in breast cancer**

**Assay**	**MammaPrint**	**Oncotype DX**	**PAM50**
**Number of genes**	**70**	**21**	**50 + 5 control genes**
Sample	Tissue core of fresh specimens preserved in RNA later or fresh-frozen tissue	Formalin-fixed, paraffin- embedded tissue, or fresh-frozen tissue	Formalin-fixed, paraffin- embedded tissue, or fresh-frozen tissue
Technique	DNA microarray	Quantitative PCR	Quantitative PCR and nCounter technology
Study population	Patients <61 years, with T1-T2, N0 disease (largely ER-positive)	Patients with ER-positive, node-negative disease	Patients with stage I to III breast cancer
Output	Low- or high-risk patients	Recurrence score: low, intermediate, or high	Risk of recurrence: low, medium, or high
Guidelines	FDA-approved	National Comprehensive Cancer Network, American Society of Clinical Oncology	FDA-cleared
Clinical applications	Accurate and reproducible representation of BC biological features [7]. Overall risk assessment of BC recurrence [8]. BluePrint and MammaPrint improve stratification of patients in the neoadjuvant setting [14,16].	Accurate and reproducible representation of BC biological features [7]. Overall risk assessment of BC recurrence [8]. Prognostic role in tamoxifen-treated patients with positive nodes [13]. Prediction of CMF efficacy in ER-positive, node-negative BC patients [13]. Prediction of FAC efficacy in ER-positive, node-positive BC patients [7].	Accurate and reproducible representation of BC biological features [7]. Categorization of tumors into the four intrinsic subtypes [8,9]. Prediction of DFS and OS [9]. Estimate of the risk of relapse at 5 to 15 years in ER-positive, node-positive and -negative BC [10]. Prediction of benefit of tamoxifen in pre-menopausal patients [9].

BC, breast cancer; CMF, cyclophosphamide, methotrexate, and 5-fluorouracil; DFS, disease-free survival; ER, estrogen receptor; FAC, fluorouracil, doxorubicin, and cyclophosphamide; FDA, US Food and Drug Administration; OS, overall survival; PAM50, Prediction Analysis of Microarray; PCR, polymerase chain reaction.

## Genomic aberrations in breast carcinogenesis as therapeutic targets

### Luminal subtypes and the PI3K/AKT/mTOR pathway

Luminal subtypes are characterized by the expression of ER and represent a heterogeneous category in terms of gene expression and clinical outcomes. The principal characteristic of this group is the luminal expression signature, composed of *ESR1*, *GATA3*, *FOXA1*, *XBP1*, and *cMYB*. The most frequent mutations in the luminal A subtype are *PIK3CA* (45%), *MAP3K1* and *GATA3* (13% each), *TP53* (12%), and *CDH1* (9%). The most frequent mutations in luminal B tumors are *TP53* and *PIK3CA* (29% each), *GATA3* (13%), and *TTN* (12%) gene mutations (Table 3) [17,18]. In addition to *TP53* mutations, frequently within luminal B subtypes, several other events may intervene in other steps of the same pathway, including *ATM* loss and *MDM2* amplification.

**Table 3:** **The most frequently mutated genes in each molecular subtype** [17,18]

**Luminal A**		**Luminal B**		**HER2-enriched**		**Basal-like**	
*PIK3CA*	45%	*PIK3CA*	29%	*TP53*	72%	*TP53*	80%
*MAP3K1*	13%	*TP53*	29%	*PIK3CA*	39%	*TTN*	19%
*GATA3*	13%	*GATA3*	13%	*MUC16*	14%	*USH2A*	11%
*TP53*	12%	*TTN*	12%	*LRP1*	8%	*FLG*	7%
*CDH1*	9%	*RYR2*	7%	*ERBB3*	8%	*MUC16*	7%
*TTN*	9%	*RELN*	5%	*DNAH11*	8%	*PIK3CA*	7%
*MLL3*	7%	*FAT3*	5%	*LRP2*	8%	*MUC17*	6%
*MAP2K4*	6%	*MLL3*	5%	*TTN*	8%	*DNAH7*	5%
*NCOR1*	5%	*MUC16*	5%	*ATP1A4*	7%	*FAT3*	5%
*AKT1*	4%	*KCNB2*	4%	*KIAA1109*	7%	*SYNE1*	5%
*PTEN*	4%	*MAP3K1*	4%	*CACNA1E*	7%	*DST*	5%

HER2, human epidermal growth factor receptor 2.

**Figure 1:**
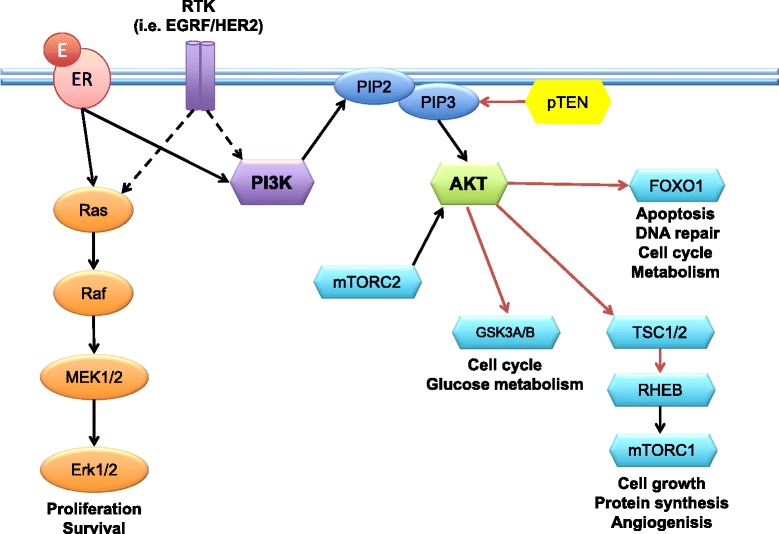
The PI3K/AKT/mTOR and the RAS/RAF/MEK/MAPK pathways. Phosphoinositide 3-kinase (PI3K) is a cytoplasmic lipid and protein kinase recruited to the membrane by activated growth factor receptors, including human epidermal growth factor receptor 2 (HER2), epidermal growth factor receptor (EGFR), and insulin-like growth factor 1 (IGF-1R). PI3K phosphorylates the 3′-hydroxyl group of phosphoinositides to produce phoshatidylinositol-3,4,5-trisphosphate (PIP3), which is a second messenger that signals through AKT to activate several enzymes, kinases, and transcription factors, including mammalian target of rapamycin (mTOR). The RAS/RAF/MEK/MAPK pathway converges with the PI3K/AKT pathway and is now recognized as an alternative in mTOR activation. On the other hand, phosphatase and tensin homolog (PTEN) catalyzes PIP3 dephosphorylation, acting as a negative regulator of its activity. In parallel with activation of growth factor receptors, estrogens can activate nuclear estrogen receptors (ERs) (genomic pathway) or ERs on the membrane (non-genomic pathway). Membrane-associated ER binds to PI3K and activates molecules such as AKT and RAS, crosstalking with the growth factor signaling pathways. Erk1/2, extracellular-signal-regulated kinase 1/2; FOXO1, Forkhead box protein O1; MEK1/2, MAPK/Erk kinase 1/2; PIP, phosphatidylinositol phosphate; Raf, murine sarcoma viral oncogene homolog; Ras, rat sarcoma viral oncogene homolog; RHEB, Ras homolog enriched in brain; RTK, receptor tyrosine kinase; TSC1/2, tuberous sclerosis proteins 1 and 2.

The PI3K/AKT/mTOR pathway and its crosstalk with the RAS/RAF/MEK/MAPK pathway play a crucial role in cancer cell growth, survival, differentiation, and proliferation (Figure 1). Moreover, the PI3K/AKT/mTOR pathway participates in the complex control of cellular energy, glucose metabolism, senescence and angiogenesis, and in ER-positive BC cells promotes ER transcriptional activity. The protein kinases involved in these pathways represent attractive and promising drug targets for BC treatment, and several molecules have already been developed in pre-clinical and clinical trials.

Although anti-estrogen therapies have been shown to reduce the risk of disease recurrence in ER-positive BC, a significant number of patients express *de novo* or acquired resistance to endocrine therapy. To date, multiple mechanisms responsible for endocrine resistance have been described, including the amplification or overexpression of the *HER2* proto-oncogene and the hyperactivation of the PI3K/AKT/mTOR pathway [19]. The most common mutations or amplifications in the PI3K/AKT/mTOR pathway affect the genes encoding the PI3K catalytic subunits (*PIK3CA*, *PIK3CB*), PI3K regulatory subunit (*PIK3R1*), receptor tyrosine kinases (*HER2, FGFR1*), K-Ras, PI3K effectors (*AKT1, AKT2, PDK1*), and loss of *PTEN* and *INPP4B* [20]. The activation of this parallel pathway may provide alternative proliferation and survival stimuli to cancer cells, even in the presence of inhibition in the ER pathway. On this basis, recent clinical trials investigated simultaneous targeting of the PI3K/AKT/mTOR and ER pathways. In particular, the mTOR inhibitor everolimus (Afinitor) in combination with the aromatase inhibitor exemestane was shown to improve survival in patients with metastatic ER-positive BC after progression on prior endocrine treatment [21]. Other mechanisms of resistance to endocrine therapies include the dysregulation of normal cell cycle control. In particular, cyclin D1 and cyclin-dependent kinase (CDK) 4/6 cooperate to phosphorylate and inactivate the retinoblastoma tumor-suppressor protein (RB), allowing cell cycle progression even in BC cells with efficient inhibition of ER [22]. Recently, the final results of the phase II PALOMA-1 trial (NCT00721409) reported a statistically significant improvement in progression-free survival (PFS) with the addition of a specific inhibitor of CDK 4/6 activity (palbociclib) to letrozole compared with letrozole alone (20.2 versus 10.2 months). The overall survival (OS) analysis showed a trend in favor of combined treatment but was not statistically significant [23]. On these bases, palbociclib and other CDK 4/6 inhibitors, including LEE011 and abemaciclib, are currently under investigation in several phase III clinical trials.

Genetic mutations and major structural defects in the DNA strands may permanently compromise gene function. In contrast, epigenetic aberrations can keep the gene structure intact and can be partially or completely reverted, restoring the original gene conformation. These transient modifications are dynamically established by enzymes which respond to intrinsic and extrinsic stimuli, and include methylation, histone acetylation, phosphorylation, ubiquitination, citrullination, sumoylation, and ADP ribosylation. In the last decade, several therapies targeting epigenetic modifying enzymes have been developed, and the FDA has approved azacitidine and decitabine for high-risk myelodysplastic syndrome and two histone deacetylase (HDAC) inhibitors (vorinostat and romidepsin) for cutaneous T-cell lymphoma [24]. For what concerns BC, recent research highlighted that DNA methylation may influence the tumor receptor status. In particular, ER positivity is linked to the methylation of specific genes, including *RASSF1A*, *CCND2*, *GSTP1*, and *TWIST*. Vesuna and colleagues [25] reported that *TWIST*, a gene overexpressed in high-grade BC, promotes downregulation of ER through the recruitment of HDAC1 and reduced expression of ER through the recruitment of DNMT3B. On this basis, azacitidine and valproic acid (HDAC inhibitor) have been investigated in this setting and showed the capability of partially restoring ER expression [25]. Moreover, in the ENCORE 301 study (NCT00676663) [26], entinostat was shown to restore sensitivity to hormonal therapy and to improve PFS and OS when given in combination with exemestane in patients with ER-positive advanced BC resistant to previous aromatase inhibitors. On the basis of this phase II trial, entinostat received a Breakthrough Therapy designation from the FDA and was included in the phase III E2112 trial (NCT02115282) that started at the beginning of 2014.

### HER2-positive breast cancer

DNA amplification of *HER2* gene is considered the main mechanism of HER2 protein overexpression in HER2-positive tumors. This subtype of tumors represents 20% to 25% of all BC, shows HER2 protein overexpression by IHC/FISH, and has been associated with poor disease-free survival rates and increased chemo-resistance. Within this clinically defined group, at least two different molecular subtypes have been identified. About 50% of clinically HER2-positive tumors express the HER2-enriched mRNA subtype, whereas the rest express predominantly the luminal mRNA subtype. HER2-enriched tumors show significantly higher expression of several receptor tyrosine kinases, including FGFR4, epidermal growth factor receptor (EGFR), HER2 itself (80%), and genes within the HER2 amplicon (that is, *GRB7*). On the other hand, the luminal mRNA subtypes show higher expression of the luminal signature, including *GATA3*, *BCL2*, and *ESR1*. In HER2-enriched tumors, the main somatic mutations were *TP53* mutations (72%) and *PIK3CA* mutations (39%), whereas *GATA3* mutations were observed only in luminal subtypes [17,20].

The HER family consists of four human EGFRs. Each member of the HER family has identified ligands, with the exception of HER2. Ligand binding to extracellular domains promotes homo- or hetero-dimerization of these receptors and promotes their intrinsic tyrosine kinase activity. In this way, the dimerization triggers various intracellular signaling pathways, including the PI3K/AKT/mTOR, RAS/RAF/MEK/MAPK, and STAT pathways. The development of trastuzumab has significantly improved the prognosis of patients with HER2-positive BC, but unfortunately a number of patients still develop resistance and progressive disease. Several mechanisms of resistance to trastuzumab have been identified: impaired access to HER2 by expression of extracellular domain-truncated HER2 (p95 HER2) or overexpression of MUC4; alternative signaling from IGF-1R, other HER family members, or MET; loss of downstream controllers (PTEN and p27); and activation of downstream signaling pathways (PI3K-Akt, MEK, MAPK, and mTOR) [27].

In the last few years, several therapeutic strategies for overcoming trastuzumab resistance have been developed, including lapatinib, pertuzumab, and trastuzumab-DM1 (Table 4). These agents act by different mechanisms on the HER2 protein and, in pre-clinical and clinical models, have been shown to be effective even in trastuzumab-resistant cells [28]. Future research should be directed to identify biomarkers that could help to choose which HER2-targeted therapy should be preferred. For instance, lapatinib is a dual HER1 (EGFR) and HER2 tyrosine kinase inhibitor and acts on the intracellular ATP-binding site of the kinase domain of the receptor. Additional studies are needed to clarify whether tumor cells with extracellular domain-truncated HER2 or with alternative signaling from EGFR could benefit from the mechanism of action of lapatinib. In these cases, reverse-phase protein microarray (RPPA) and next-generation sequencing (NGS) tests could be used as clinical diagnostic assays to study HER2 protein status or alterations within the signaling pathways, guiding treatment decision-making. Moreover, in a subanalysis of the EMILIA trial (NCT00829166) [29], HER2 mRNA analysis by quantitative reverse transcriptase-polymerase chain reaction and the PIK3CA mutational status were analyzed on tumor tissues. Cases expressing higher levels of HER2 mRNA showed better OS from T-DM1, whereas PIK3CA mutational status was not demonstrated to predict outcomes in patients receiving T-DM1. On the other hand, in the group receiving lapatinib and capecitabine, HER2 mRNA was not demonstrated to influence outcomes, whereas patients with mutated PIK3CA status showed shorter median PFS and OS [30]. In conclusion, future research is necessary to investigate BC cells at the molecular level in order to select genomic and proteomic tools that could guide targeted treatment choices.

**Table 4:** **Biologic anti-HER2 agents approved for breast cancer treatment**

**Agent**	**Approval**	**Mechanism of action**	**Indications**
Trastuzumab	1998	Humanized monoclonal antibody against the extracellular domain of HER2. It triggers HER2 internalization and degradation.	Adjuvant BC
			Metastatic BC
			(from the first line)
Lapatinib	2006	Dual inhibitor of the intracellular tyrosine kinase domains of both HER1 (EGFR) and HER2.	Metastatic BC
			(after prior anthracycline, taxane, and trastuzumab)
Pertuzumab	2012	Humanized monoclonal antibody against the extracellular dimerization domain of HER2. It blocks the heterodimerization of HER2 with other HER family.	Neoadjuvant BC
			(with trastuzumab and docetaxel in locally advanced, inflammatory, or early stage BC -either >2 cm or node-positive)
			Metastatic BC
			(with trastuzumab and docetaxel for first-line therapy)
T-DM1	2013	Trastuzumab-like activity.	Metastatic BC (after the first line or in first line if trastuzumab-resistance)
		Targeted intracellular delivery of cytotoxic emtansine.	

BC, breast cancer; EGFR, epidermal growth factor receptor; HER2, human epidermal growth factor receptor 2.

Other potential strategies for overcoming trastuzumab resistance include PI3K/AKT/mTOR pathway inhibitors, HER2 vaccines, inhibitors of alternative signaling molecules (IGF-1R and MET), ertumaxomab, and defucosylated trastuzumab. Moreover, dual inhibition of HER2, combining more monoclonal antibodies against the HER2 extracellular domain, could be another effective approach to treat trastuzumab-refractory HER2-positive tumors.

In the last few years, the inhibition of heat shock protein 90 (Hsp90) has emerged as an attractive approach in order to overcome trastuzumab resistance. Hsp90 is a ubiquitous molecular chaperone fundamental for correct folding and maturation of numerous cellular proteins. Inhibition of the Hsp90 chaperone cycle leads to protein ubiquitination and subsequent degradation by the proteasome. Because Hsp90 client proteins are frequently products of oncogenes, Hsp90 may represent an important target in cancer therapy. Moreover, because Hsp90 protects cells from stress-induced damage, Hsp90 inhibitors could sensitize cells to cytotoxic agents and radiation therapy. Hsp90 inhibitors are currently under investigation in several clinical trials and have shown early promising results in defined molecular subgroups of solid tumors such as the HER2-positive BC [31]. HER2 is one of the most sensitive client proteins to Hsp90 inhibition and thus in the absence of Hsp90 activity HER2 is subject to proteolysis, and drugs that target HER2 can be more effective. To date, promising results in terms of objective tumor response have been observed with tanespimycin in combination with trastuzumab in patients progressing on trastuzumab (NCT00773344) [32] and more recently with single-agent ganetespib in trastuzumab-refractory HER2-positive tumors and TNBC (NCT01677455) [33].

### Subtypes of triple-negative breast cancer and p53 mutations

TNBC constitutes about 15% to 20% of all BC and is clinically defined by the absence of ER and PR positivity and the lack of HER2 overexpression by IHC. This heterogeneous group of tumors is more aggressive, with higher rates of relapse and worse OS. The basal-like tumor represents a specific group characterized by the expression of genes found in normal basal/myoepithelial breast cells, including high-molecular-weight basal cytokeratins (CK5/6, CK14, and CK17) or EGFR or both. For years, the relationship between TNBC and basal-like tumors has been controversial. However, not all TNBCs are identified as basal-like tumors by gene expression, and not all basal-like tumors are clinically TNBC; therefore, these two terms may not be considered synonymous. In 2011, Lehmann and colleagues [34] identified six different subtypes within TNBC by performing RNA microarray analyses: two basal-like (BL1 and BL2), an immunomodulatory (IM), a mesenchymal (M), a mesenchymal stem-like (MSL), and a luminal androgen receptor (LAR) subtype. BL1 and BL2 subtypes heavily express cell cycle and DNA damage response genes (*ATR/BRCA*), and representative cell lines respond particularly to anti-mitotic and DNA-damaging agents, such as platinum agents. In particular, the poly (ADP-ribose) polymerase (PARP) family of enzymes, the DNA damage response kinase ATR and its effector kinases CHEK1/2, and the regulator of the G_2_ checkpoint WEE1 kinase represent attractive pharmacological targets for radiosensitization and chemosensitization in these cells. Several ATR, CHEK1/2, and WEE1 inhibitors are currently under evaluation in phase I/II trials in TNBC, and a number of PARP inhibitors are already included in phase III studies. On the other hand, the IM subtype is highly enriched in immune cell signaling, but it is still unclear whether this genetic profile is reflective of cancer cells or stromal immune infiltrate. M and MSL subtypes express genes involved in epithelial-mesenchymal transition (EMT), motility, and cell differentiation pathways, and its representative cell lines respond to PI3K/mTOR inhibitors and dasatinib. Moreover, the MSL subtype is enriched for mesenchymal stem cell-associated genes. Finally, the LAR subtype expresses luminal signature and androgen receptor (AR) signaling. LAR subtypes are associated with better OS, and a recent phase II trial of bicatulamide in AR-positive TNBC (NCT00468715) reported a 6-month clinical benefit rate of 19% with a median PFS of 12 weeks, showing proof of principle for the effectiveness of minimally toxic androgen blockade in this subgroup of patients [35]. Currently, enzalutamide and abiraterone are also under evaluation in phase II studies in this select subset of patients.

Previous evaluations of gene expression profiles in TNBC had led to the identification of another subgroup named claudin-low and characterized by the absent expression of luminal differentiation markers, high enrichment for EMT markers, immune response genes, and cancer stem cell (CSC)-like features [36]. However, Lehmann and Pietenpol [37] observed that most of the tumors classified as claudin-low are composed of M and MSL subtypes, and concluded that the classification into basal-like and non-basal-like subtypes oversimplifies the molecular heterogeneity of TNBC. A comprehensive classification of TNBC in molecular subtypes has the potential to guide treatment decision-making and future clinical trials investigating targeted therapies.

*TP53* mutations are the most frequent clonal aberrations in basal-like tumors (80%), but the loss of TP53 function, through gene mutations or dysfunctions in the TP53 pathway, occurs within almost all basal-like tumors. *TP53* is a tumor-suppressor gene that, after the activation by oncogenic stress signals, promotes either cell cycle arrest and DNA repair or cell apoptosis. The activity of *TP53* is achieved by downstream targets (p21), indirect targets (PTEN), cell cycle regulation proteins (Chk1 and Chk2), and DNA repair proteins (PARP-1 and BRCA-1). Moreover, the TP53 pathway is influenced by prolyl isomerise 1, which is responsible for post-translational modifications and histone acetylation, which applies epigenetic modifications to the *TP53* gene. Mutated *TP53* represents a potential target for TNBC treatment but this approach remains a challenge on several fronts since our knowledge of the TP53 pathway is still largely incomplete and the heterogeneity of TNBC comprises different behavior even among the subgroups showing aberrant TP53 function [38]. In addition to loss of TP53, loss of RB1 and BRCA1 functions are common basal-like features. *PIK3CA* is the next most commonly mutated gene (9%). However, interferences in PI3K pathway activity are even more frequent in basal-like tumors and include loss of *PTEN* and *INPP4B*. Furthermore, in basal-like cancers, many of the elements of the PI3K/AKT/mTOR and RAS/RAF/MEK/MAPK pathways were amplified, including PIK3CA (49%), KRAS (32%), BRAF (30%), and EGFR (23%) [17].

To date, on these molecular bases, many agents have been included in clinical trials for patients with TNBC. In particular, cabozantinib, a MET-, VEGFR2-, and RET-targeted tyrosine kinase inhibitor, is under evaluation in a phase II trial (NCT01738438). OTX015 and TEN-010 are bromodomain and extra-terminal domain (BET) bromodomain inhibitors that can inactivate the expression of oncogenes such as *Myc*. These novel epigenetic modulators of gene expression are included in phase I trials for patients with TNBC and solid tumors.

## Breast cancer heterogeneity

BC represents a heterogeneous disease at the population and single-cell level. Cancer cells within the same tumor can exhibit different genotypes as well as phenotypes. In the last decade, the development of NGS methods provided fundamental insight into the genetic intra-tumor heterogeneity, and different models have been proposed to explain the origins of this phenomenon. The first model, proposed by Nowell in 1976, suggests that tumor masses are caused by the expansion of one (monoclonal) or multiple (polyclonal) cellular clones [39]. In the CSC model, some precursor cells give rise to a different subpopulation of cells within the tumor with a hierarchical arrangement. Lastly, in the mutator hypothesis, tumor mass develops because of the gradual and random accumulation of genetic mutations, causing a wide range of diversity [40].

In the last few years, a plastic CSC model has been developed. The emerging CSC model suggests that cancer initialization, progression, and metastasization are driven by a specific subpopulation of tumor cells (named CSCs) that express two fundamental characteristics: the capacity for self-renewal and the ability to efficiently reconstitute differentiated tumors. These cells are associated with phenotypic plasticity with the acquisition of peculiar mesenchymal characteristics during specific phases of the metastatic process. The EMT is a latent embryonic process that, when aberrantly activated in cancer cells, promotes the reprogramming of epithelial cancer cells toward a mesenchymal motile phenotype with migratory and invasive capacities and thus metastatic potential [41]. EMT is a highly dynamic process that is represented by several steps and multiple intermediate states and that contributes to increase the cancer heterogeneity and plasticity. The transition is induced by several extracellular stimuli, including different growth factors (EGF, PDGF, and TGFβ), Hedgehog (Hh), Wnt/β-catenin, Notch, and components of the extracellular matrix and cellular stress conditions such as hypoxia. In addition to migratory and invasive capabilities, induction of EMT showed the ability to generate cells that exhibit molecular and functional stem-like characteristics, leading to the expression of stem-cell markers such as the CD44^+^/CD24^−^ antigenic phenotype combined to the expression of ALDH-1. According to this hypothesis, tumors can originate from the transformation of normal adult tissue stem cells or from more differentiated progenitors that have acquired stem-like capabilities [42]. On the other hand, disseminated cancer cells can perform a mesenchymal-epithelial transition reverting to an epithelial phenotype in order to adhere and proliferate at distal sites [43].

**Figure 2:**
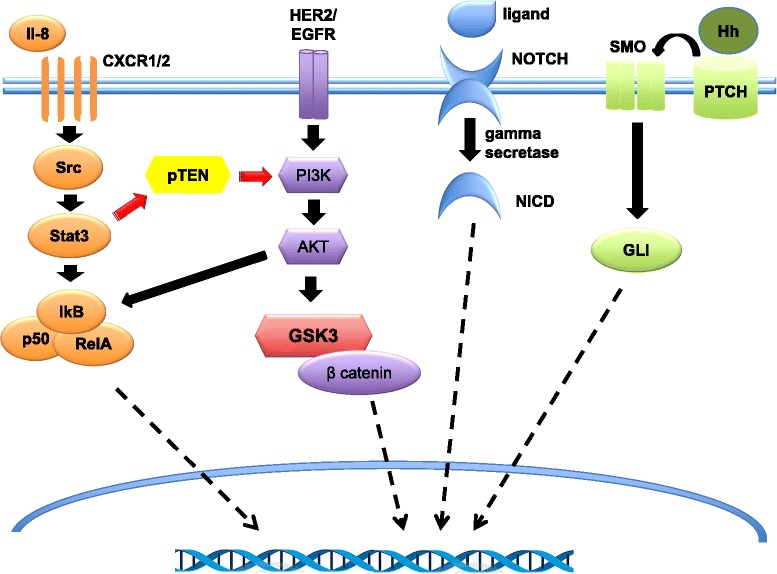
Molecular pathways regulating breast cancer stem cells (CSCs). Akt represents a central hub in the Wnt/β-catenin and phosphoinositide 3-kinase (PI3K) signaling pathways. Upstream of Akt is the phosphatase and tensin homolog (PTEN) tumor suppressor. Loss of PTEN results in Akt activation and thus the activation of the Wnt/β-catenin pathway through the Akt-mediated phosphorylation of glycogen synthase kinase 3-beta (GSK3-β) and nuclear translocation of β-catenin. Through autocrine, juxtacrine, and paracrine mechanisms, secreted Hedgehog (Hh) interacts with the 12 trans-membrane Patched 1 (PTCH) receptor, de-repressing the 7 trans-membrane Smoothened (SMO) protein and allowing its translocation to the cilia. Activated SMO promotes a signaling cascade resulting in activation of the GLI transcription factors and thus in upregulation of genes that regulate cellular differentiation, proliferation, and survival. Four different Notch receptors (Notch 1, Notch 2, Notch 3, and Notch 4) interact with five ligands (Delta-like 1, Delta-like 3, Delta-like 4, Jagged 1, and Jagged 2) expressed on neighboring cells. The interaction between ligand and the extracellular domain of Notch receptor triggers the cleavage by gamma-secretase and the release of the Notch intracellular domain (NICD), which translocates into the nucleus and associates with transcription factors regulating Notch target genes expression. The interaction between CXCR1/2 and interleukin (IL)-8 increases CSC self-renewal. HER2 regulates CSCs through the activation of the Wnt/β-catenin pathway, and loss of PTEN results in the downstream activation of the Wnt/β-catenin signaling. The activation of an inflammatory loop involving IL-6 and IL-8 has been shown to determine PTEN suppression and thus the resistance to HER2-targeting agents. IkB, kinase B inhibitor; p50, protein 50; Src, rous sarcoma oncogene cellular homolog; Stat3, signal transducer and activator of transcription 3.

This clonal heterogeneity confers greater resistance to selective environmental pressures and mostly to chemotherapy and radiation therapy [44]. As a matter of fact, high-grade tumors, typically the basal-like and triple-negative subtypes, showed the highest levels of genetic diversity and the highest number of cells that express molecular signatures characteristic of EMT, and thus they are associated with worse clinical outcomes [45]. On this basis, CSCs may represent an attractive and promising target for the development of future drugs. In particular, novel therapeutic options are directed to inhibit the pathways regulating the growth, survival, and self-renewal of CSCs, including the Notch, Wnt/β-catenin, Hh, and PI3K pathways (Figure 2) [46].

Akt represents a central hub in the Wnt/β-catenin and PI3K signaling pathways. Pharmacological inhibition of Akt may reduce tumor growth and regulate malignant stem cells [47]. Notch signaling is an evolutionarily conserved pathway that mediates communication between adjacent cells and plays both oncogenic and tumor-suppressor roles in various malignancies. Gamma-secretase inhibitors represent an attractive therapeutic option for the inhibition of the Notch pathway and are currently under investigation in clinical trials for Alzheimer’s disease, T-cell acute lymphoblastic leukemia, and BC [46]. Furthermore, Harrison and colleagues [48] observed that Notch4 signaling activity was notably higher than Notch1 signaling activity in stem cell-enriched cell populations, suggesting that selective inhibition of Notch4 could be more effective and potentially less toxic.

Finally, several cytokines have been shown to be involved in the maintenance of CSCs, including interleukin (IL)-6 and IL-8. In particular, CXCR1/2, the receptor of IL-8, has been found to be overexpressed in cancer cells expressing the stem cell marker ALDH [49]. The interaction between CXCR1/2 and IL-8 increases CSC self-renewal; thus, CXCR1/2 blockade may represent another attractive target. Moreover, Singh and colleagues [50] recently observed an interaction between the CXCR1/2 signaling and the HER2 pathway, suggesting that HER2-blocking agents might synergize with CXCR1/2 inhibitors in targeting the CSCs. The clinical evaluation and incorporation of stem-cell targeted therapies in the future management of patients with BC present challenges but also the opportunity to modify the natural history of the most aggressive forms of this disease.

## Immune pathway

The immune system could play a role in the effectiveness of conventional anticancer treatments. Higher levels of tumor-infiltrating lymphocytes (TIL) have been associated with a higher rate of pCR after NACT, and post-chemotherapy TIL presence has been linked with better outcomes in patients who did not obtain pCR after neoadjuvant paclitaxel. Especially in TNBC, high levels of TIL in residual disease after NACT represent a strong prognostic factor and thus may help to identify patients who, despite residual disease, will have good outcome. Moreover, the conversion from originally low-TIL tumors into high-TIL tumors after NACT has been associated with a significant improvement in OS, suggesting that chemotherapy could induce an antitumor immune response [51,52]. Also in the adjuvant setting, high TIL level has been significantly associated with decreased distant recurrence rates in primary TNBC treated with conventional adjuvant chemotherapy [53].

The involvement of the immune system has been confirmed even in trastuzumab antitumor activity. Recent evidence has demonstrated that TIL could predict efficacy of trastuzumab, since they increase its benefit in HER2-positive tumors [53]. These results suggest that immune infiltration may represent a useful parameter to stratify patients eligible for treatments, provide evidence for the development of immune-mediated therapies in BC, and may represent a surrogate for detection of NACT efficacy.

## Conclusions

The use of advanced diagnostics in BC has increased our understanding of disease biology and is currently applied to clinical practice. Evaluation of standard biomarkers (ER, PR, and HER2) in primary BC can be supplemented by molecular profiling with significant information on disease subtyping, which constitutes prognostic and predictive data that can help in treatment planning. Moreover, the application of NGS and RPPA in the metastatic setting holds promise in further advancing precision medicine. Patients with a BC diagnosis can now benefit from selection of a number of novel targeted therapies that are either FDA-approved or under development and that have already translated to significant improvement in survival for this disease. Unfortunately, approximately 40,000 women a year still die of metastatic disease in the United States, suggesting that we have made limited strives in investigating and treating metastasis. The integration of CSC-targeted therapies and immune therapies in future combination regimens holds promise in addressing this unexplored area of clinical research and likely curing a larger proportion of women with metastatic BC.

## Abbreviations

AR: Androgen receptor
BC: Breast cancer
BL: Basal-like
CDK: Cyclin-dependent kinase
CSC: Cancer stem cell
EGFR: Epidermal growth factor receptor
EMT: Epithelial-mesenchymal transition
ER: Estrogen receptor
*ESR1*: Estrogen receptor 1
FDA: US food and drug administration
FISH: Fluorescence *in situ* hybridization
HDAC: Histone deacetylase
HER2: Human epidermal growth factor receptor 2
Hh: Hedgehog
HR: Hormone receptor
Hsp90: Heat shock protein 90
IHC: Immunohistochemistry
IL: Interleukin
IM: Immunomodulatory
LAR: Luminal androgen receptor
M: Mesenchymal
MSL: Mesenchymal stem-like
NACT: Neoadjuvant chemotherapy
NGS: Next-generation sequencing
OS: Overall survival
PAM50: Prediction analysis of microarray
PARP: Poly (ADP-ribose) polymerase
pCR: pathologic complete response
PFS: Progression-free survival
PR: Progesterone receptor
RB: Retinoblastoma tumor-suppressor protein
ROR: Risk of recurrence
RPPA: Reverse-phase protein microarray
RS: Recurrence score
TIL: Tumor-infiltrating lymphocytes
TNBC: Triple-negative breast cancer
